# Evaluating *Plasmodium falciparum* automatic detection and parasitemia estimation: A comparative study on thin blood smear images

**DOI:** 10.1371/journal.pone.0304789

**Published:** 2024-06-03

**Authors:** Aniss Acherar, Xavier Tannier, Ilhame Tantaoui, Jean-Yves Brossas, Marc Thellier, Renaud Piarroux

**Affiliations:** 1 Inserm, Institut Pierre-Louis d’Épidémiologie et de Santé Publique, IPLESP, Sorbonne Université, Paris, France; 2 SCAI (Sorbonne Center for Artificial Intelligence), Sorbonne Université, Paris, France; 3 Sorbonne Université, Inserm, Laboratoire d’Informatique Médicale et d’Ingénierie des Connaissances pour la e-Santé, LIMICS, Université Sorbonne Paris Nord, Paris, France; 4 AP-HP, Service de Parasitologie-Mycologie, CNR du Paludisme, Groupe Hospitalier Pitié-Salpêtrière, Paris, France; Para Federal University, BRAZIL

## Abstract

Malaria is a deadly disease that is transmitted through mosquito bites. Microscopists use a microscope to examine thin blood smears at high magnification (1000x) to identify parasites in red blood cells (RBCs). Estimating parasitemia is essential in determining the severity of the *Plasmodium falciparum* infection and guiding treatment. However, this process is time-consuming, labor-intensive, and subject to variation, which can directly affect patient outcomes. In this retrospective study, we compared three methods for measuring parasitemia from a collection of anonymized thin blood smears of patients with *Plasmodium falciparum* obtained from the Clinical Department of Parasitology-Mycology, National Reference Center (NRC) for Malaria in Paris, France. We first analyzed the impact of the number of field images on parasitemia count using our framework, MALARIS, which features a top-classifier convolutional neural network (CNN). Additionally, we studied the variation between different microscopists using two manual techniques to demonstrate the need for a reliable and reproducible automated system. Finally, we included thin blood smear images from an additional 102 patients to compare the performance and correlation of our system with manual microscopy and flow cytometry. Our results showed strong correlations between the three methods, with a coefficient of determination between 0.87 and 0.92.

## Introduction

Malaria is a significant public health concern, particularly in areas where resources are limited. According to the World Health Organization (WHO), there were approximately 241 million cases and 627,000 deaths from malaria worldwide in 2021 [1]. This represents 14 million more cases and 69,000 more deaths than in 2020. Malaria is characterized by the presence of protozoan parasites called *Plasmodium* transmitted by infected female Anopheles. There are 5 different species of parasites infecting humans, *Plasmodium vivax*, *Plasmodium ovale*, *Plasmodium malariae*, *Plasmodium knowlesi* and *Plasmodium falciparum*. *Plasmodium falciparum* is by far the most common and the most lethal. Malaria is a medical emergency that requires immediate diagnosis to prevent severe cases and deaths [2].

Several diagnostic approaches have been proposed. The simplest techniques to implement, particularly in low-income countries, are rapid diagnostic tests (RDTs) [3], which are based on the detection of the *P*. *falciparum* specific HRP2 protein. However, this cost-effective strategy is being questioned due to the high number of false-positive results. The HRP2 protein can be detected several weeks after recovery from a malaria attack, but, more worryingly, there is an increased risk of false-negative results due to the diffusion of parasitic clones that are mutant for the HRP2/HRP3 proteins [2]. Molecular biology techniques such as polymerase chain reaction (PCR) and loop-mediated isothermal amplification (LAMP) [4] are much more sensitive, but they are too costly and time-consuming to be implemented routinely in every setting. For the diagnosis of malaria, the recommended strategy in non-endemic countries is thick blood smear or molecular biology techniques. It’s worth noting that there may be non-endemic areas or regions within endemic countries where microscopy or RDT is used. In practice, since most laboratories do not have expertise in thick blood smears, molecular biology techniques, such as LAMP, are commonly employed. As a second step, for positive cases, a thin blood smear is systematically performed. Diagnosis of malaria is not limited to identifying the presence of the parasite; it must also answer questions such as: what species of *Plasmodium* is involved, what is the parasitemia in the case of *P*. *falciparum* malaria, and what parasite stages are present in the blood? Unfortunately, in the context of routine emergency malaria diagnosis, some diagnostic techniques such as RDTs or molecular methods may not provide complete answers to all these questions. However, through a thin smear, it is possible to determine parasitemia, identify the species, and detect parasite stages. Microscopic examination of the parasite remains a crucial aspect of malaria diagnosis. Accurately estimating parasitemia requires examining a thin blood smear, which is all the more crucial as parasitemia is a significant criterion in determining the severity of a malaria attack. In fact, a parasitemia above 4% in adults and 10% in children under 5 is among the criteria for defining severe *P*. *falciparum* infection [5]. Parasitemia is usually estimated manually using standard microscopy techniques (thick blood smear, quantitative buffy coat) [6]. However, the main drawback of this process is that it is time-consuming and subject to significant intermicroscopist variability [7]. Microscopic examination requires the mobilization of an expert microscopist 24/7 to make the diagnosis. Overall, the reliability of the diagnosis depends heavily on this human expertise, which can vary greatly from one microscopist to another. This requires maintaining the skills of the personnel and controlling the quality of the results produced.

Artificial intelligence (AI) is increasingly being used to enhance malaria diagnosis. One potential application of AI in malaria diagnosis is the use of machine learning (ML) and specifically deep learning (DL) algorithms such as CNNs to detect malaria and estimate parasitemia from a thin blood smear. DL and other AI-based approaches offer the potential for faster and more accurate estimation of parasitemia by training CNNs on a large microscopy image dataset. Several studies have examined the use of these approaches for malaria detection [8] and species and life-stage identification [9]. Other studies have compared the performance of AI-based systems with a panel of experts to demonstrate their potential [10]. The use of CNNs has been investigated to detect and count white blood cells (WBCs) and quantify parasitemia on thick blood smear images, where quantification concordance with expert microscopy reported weak correlation coefficients ranging from 0.59 to 0.86 [11]. Some studies have shown that there may be a weak correlation between manual microscopy results and estimations by the CNNs (R^2^ = 0.55) [12] or an overestimation of the model for parasite densities <1000 p/μL [13]. Another study conducted a multicenter evaluation in primarily endemic regions, assessing an ML system for species identification and parasitemia measurement, including 624 thick blood smears infected with *P*. *falciparum*. Strong concordance scores were observed between microscopy and the proposed system, with average variations of approximately ±25% [14]. These studies cited have mainly focused on the quantification of parasites from thick blood smears. In our study we use thin blood smears. Outside endemic areas, malaria is a rare disease (an estimated 5,000 cases per year in mainland France, for 50 to 100,000 diagnostic requests). As the thick blood smear is only used for this indication, it is poorly mastered by most diagnostic laboratories. The thick blood smear is therefore often replaced by a molecular biology technique (a LAMP technique for the most part). The recommended strategy outside malaria-endemic areas is to perform a thick blood smear or LAMP technique as a first-line test. If the test performed at this first line is positive, a thin blood smear is then systematically performed to provide the requesting physician with a diagnosis of the species, the parasite stages, and the measurement of parasitemia as a percentage of parasitized RBCs. It should also be pointed out that measuring the parasite load on a thick blood smear is far less accurate than on a thin smear. RBCs are lysed, young *P*. *falciparum* trophozoites are also lysed to an extent that is difficult to determine, and the estimation of the number of parasites in relation to white blood cells is very approximative. Measuring parasitemia on thick blood smears in endemic areas is particularly useful for epidemiological surveys and is much less relevant for the initial management and follow-up of treated patients.

Thus, among the few attempts devoted to the assessment of the parasitemia from thin blood smears with other techniques, traditional ML approaches and neural networks were used, such as support vector machine (SVM) and multilayer perceptron (MLP), to quantify parasitemia on preextracted features of RBCs from thin blood smear images [15]. Strong correlations were observed (R^2^ = 0.97) between manual and semiautomatic results; however, the sample size remained small, with only 9 slides and a total of 2,500 RBCs analyzed and 31 patients (19 patients with a malaria infection and 12 uninfected controls) [16]. Other studies investigated the use of artificial neural networks for the classification of preextracted features compared with commercial flow cytometry [17]. The evaluation performed on 2 mice with a follow-up of 2 weeks also showed an overcounting of parasitemia due to the presence of false-positives induced by staining artifacts. However, the sample size remains small and does not allow a precise evaluation of the correlation between the 2 techniques.

As shown above, no comprehensive study has evaluated the utilization of a DL-based system for accurate parasitemia estimation and its comparison with various manual and semi-automatic techniques using a substantial collection of thin blood smear images selected from routine patient care settings. Therefore, in this study, we aimed to evaluate the ability of our recently published DL-based framework, MALARIS, which was trained on blood cell images acquired at 500x magnification, to accurately estimate parasitemia on thin blood smear images obtained from patients diagnosed with imported malaria caused by *Plasmodium falciparum* [18]. Specifically, (1) we conducted a repeatability study to examine the evolution of the parasitemia variation as a function of the number of fields examined, (2) we conducted an experiment to highlight the variability between different microscopists regarding the measurement of parasitemia using manual techniques, and (3) we evaluated MALARIS by comparing its concordance with manual microscopy and semi-automatic techniques.

## Material and methods

The laboratory: the Parasitology-Mycology Department at the Pitié-Salpêtrière Hospital is home to the National Malaria Reference Center (NMRC). It is accredited under ISO15189 by Cofrac for all the malaria diagnostic techniques it performs on a routine basis (around 1,500 samples analyzed, including 600 as part of the NRC activity): light microscopy for thin and thick blood smears techniques, molecular biology, rapid diagnostic tests and flow cytometry.

### Dataset selection

In the clinical department of Parasitology-Mycology, National Reference Center (NRC) for Malaria, Paris, France, we randomly selected from 456 samples, 116 Giemsa-stained (RAL, France) [19], thin blood smears from patients positive for *P*. *falciparum* species. The slides made between January 2019 and August 2022 were anonymized for the study. Expert parasitemia measurements ranged from 0.15% to 26.2%. Images were captured in a JPG RGB format, on Olympus BX51 and BX41 light microscopes at 500x magnification. Both the Olympus BX51 and BX41 microscopes are equipped with the UIS2 infinite optical system, which minimizes distortion and enhances image quality. For the BX41, we used an Olympus UPlanSApo 50x lens, and for the BX51, we used a UPlanFl 50x/0.50P lens. Both are oil immersion lenses with increased numerical aperture (NA). The smartphone was attached to a WHN 10x/22 eyepiece using an Orion 05306 SteadyPix Pro adapter. To assess the repeatability of parasitemia estimation, a panel of 14 blood films was used to compare two manual measurement techniques, the standard WHO technique, and the Miller reticle technique. Then, 102 blood films were used to compare the performance of our DL-based system with both the Miller reticle and an automated flow cytometry counting method. In total, 1,160 images were captured using two different smartphones: the 12-megapixel rear camera of an iPhone 7 and a Google Pixel 6 with a dual rear camera system. The Pixel 6 included a 50-megapixel sensor and a 12-megapixel sensor for wide-angle shots. For this study, we used the Ultra-wide one: 12 MP 1/2.86″ sensor,16mm equivalent f/2.2-aperture lens. All experiments described in this study were conducted between 2019 and 2022.

### Parasitemia measurement: Leveraging the DL-based framework MALARIS with standard microscopy measurements and Miller reticle assistance

For this study, we called our pipeline MALARIS. It is based on our previous study [18], where we compared the performance of different CNN architectures, including VGG-19 [20], ResNet-50 [21], and EfficientNet-B7 [22] and we evaluated their performance and generalization on 200 slides from 50 infected and 150 uninfected patients in real-life conditions. The system can detect and classify blood cell images into *Plasmodium falciparum-infected* RBCs and uninfected blood components on thin blood smears at 500x magnification. The patient-level evaluation showed good performance scores in terms of generalization and achieved accuracy, sensitivity and specificity scores of 99.7%, 77.9% and 99.8%, respectively. In this context, we chose the highest-performing model from the EfficientNet series to classify the images into two classes: red blood cells infected by *P*. *falciparum* and uninfected blood components. To reproduce the study’s results, we have provided a Jupyter Notebook and all relevant data for parasitemia measurements. Access to this notebook, along with the MALARIS model and code for calculating parasitemia on thin blood smear images at x500 magnification, is available on our GitHub repository at https://github.com/anissacherar/MALARIS.git. It’s important to note that access to the complete image dataset is restricted in compliance with patient confidentiality regulations. Requests for access to this data should be directed to the relevant department at AP-HP Pitié Salpêtrière via email at secretariat.parasitologie.psl@aphp.fr.

**Fig 1:**
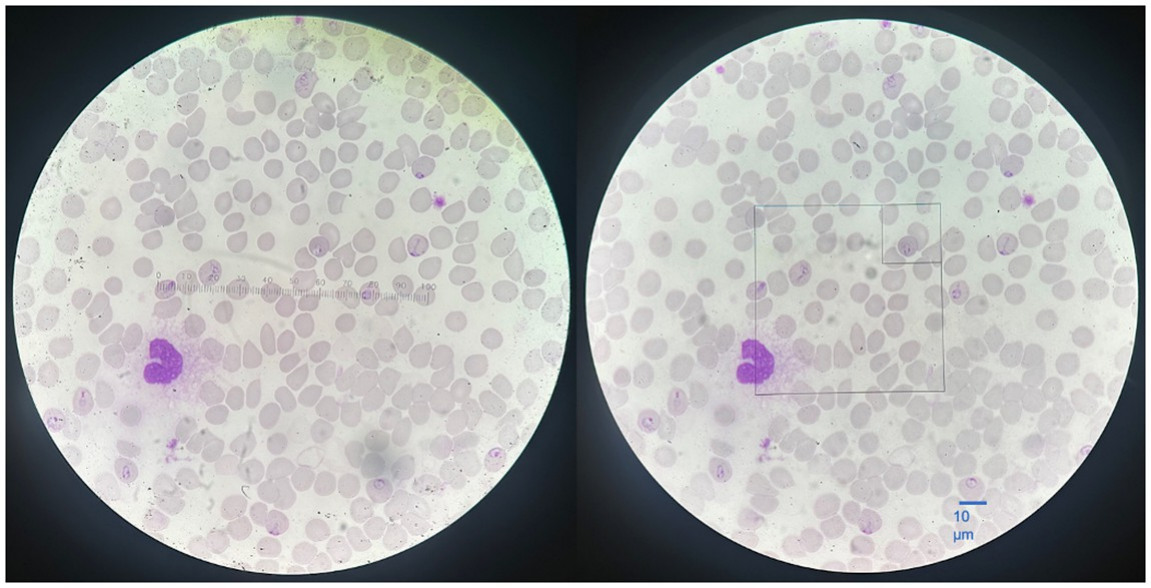
Image of a *Plasmodium falciparum*-infected thin blood smear at 1000x magnification, both without and with the Miller reticle. At 1000x magnification, one graduation = 1 micrometer. The average size of RBCs is 7μm (ranging from 5 to 10).

The standard measurement of parasitemia (S1 Text) was calculated by dividing the total number of infected red blood cells (excluding gametocytes) by the total number of red blood cells counted or estimated in the examined microscopic fields. To estimate parasitemia on thin blood smears viewed under light microscopy, we followed a standard operating procedure adapted from the WHO guidelines [23]. At 1000x magnification, we manually counted all infected RBCs in the numerator while the denominator involved counting the total number of RBCs in a representative field (Fig 1) to estimate the global denominator. The process was then repeated by counting infected RBCs in each subsequent field. The WHO recommends examining 5,000 RBCs, which is approximately 20 fields, to estimate parasitemia. The denominator, the total number of RBCs examined, was estimated by multiplying the number of fields by the number of RBCs in a representative field.

The Miller disc or reticle (Fig 1) count method uses the ratio of two squares of different sizes to count a large number of cells, especially when the distribution from one field to another is highly variable. The Miller reticle was originally used in the field of hematology, specifically for reticulocyte counting [24], where it was shown to reduce imprecision when counting manually. For the measurement of malaria parasitemia, the procedure involves counting the total number of RBCs in the small square, estimating their number in the large square using a 1:10 ratio, and then counting the total number of infected RBCs in the large square. This results in a more accurate estimation of the denominator in each field examined (large square). To estimate parasitemia on thin blood smears viewed under light microscopy, we followed a standard operating procedure.

### Parasitemia measurement using flow cytometry: Analysis of patient RBCs with anti-RESA polyclonal antibody and SYBR Green I dye

For parasitemia measurement using flow cytometry, red blood cells (RBCs) from the patient (1 × 10^7^) were washed with 200 μL of TBH buffer containing 0.5x HBSS (Life Technologies Limited. Paisley, UK), 0.5x VERSOL^®^ (AGUETTANT. Lyon France), and 50 mM Tris HCl (pH of 7.0). Then, the cells were treated with 1% glutaraldehyde in PBS for 1 minute at room temperature and washed four times. Next, the cells were incubated at 37°C with shaking for 30 minutes with 25 μg/mL RESA anti-repeat peptide polyclonal antibody that was developed in our lab and then washed three times with 200 μL PBS. The cells were then resuspended in 200 μL of AlbuMaxTM II 1% (Life Technologies NZ Ltd Auckland, USA), and 0.1% TRITON X100 in PBS containing 10 μg/mL Pacific BlueTM conjugated goat anti-rabbit IgG-(H+L) fragment (INVITROGEN. Oregon, USA) and 4x SYBRTM Green I (INVITROGEN. Carlsbarg, USA). After 30 minutes of incubation at 37°C with shaking, the cells were washed three times with 200 μL PBS before being subjected to flow cytometry assay using CytoFlex (Beckman Coulter. Pasadena, USA). RBCs were gated based on their forward and side scatter characteristics, and 50,000 events were acquired. *P*. *falciparum*-infected RBCs were RESA positive and SYBR positive, whereas noninfected RBCs were RESA negative and SYBR negative. The events were analyzed using CytExpert software.

### Manual techniques as a source of variability for parasitemia

We first examined the repeatability of two manual techniques for estimating parasitemia: the standard measurement and a measurement assisted by the Miller reticle. The validation of the method using the Miller cell for estimating parasitemia was conducted at the parasitology lab of the Pitié-Salpêtrière Malaria NRC (National Reference Center) in France. To confirm its effectiveness, we selected 14 infected patients, with one smear per patient and the parasitemia of each smear was estimated by 10 independent microscopists, including residents, medical biologists, and laboratory technicians who are part of the parasitology lab. We evaluated the variation of the parasitemia estimation of the microscopists by calculating the Relative Standard Deviation (RSD) (S2 Text). This allowed us to analyze the variability and repeatability of each technique for each patient, considering the differences among operators. The standard measurement corresponds to an estimation of parasitemia based on 20 fields at 1000x magnification, while the Miller reticle-assisted technique corresponds to an estimation based on 50 fields.

### Parasitemia repeatability using MALARIS: The impact of the number of fields

Here, we examined the variability and repeatability of parasitemia estimation using MALARIS. We utilized a batch of 14 patients, from whom we obtained 10 microscopic field images each. To assess the potential variations resulting from selecting different fields (repeatability), we conducted multiple comparisons between two experiments using two distinct sets of n fields (k between 1 and 5). We evaluated the repeatability and the impact of the number of fields on each parasitemia estimation with the traditional relative standard error (RSE) between two estimates on all patients. This provides an indication of the optimal number of fields to choose to have a reliable estimate of the parasitemia (S3 Text).

### MALARIS vs. manual and semiautomatic methods, including Miller reticle and flow cytometry

We conducted a comparison between our MALARIS system and two other methods, the Miller disc and flow cytometry. To evaluate the correlation of parasitemia estimation, we analyzed 102 additional patients and calculated the coefficient of determination (R^2^) for each pair of methods. For each patient, the MALARIS system analyzed 5 images captured at 500x magnification, i.e., a total of over 5,000 erythrocyte cells; the Miller reticle method is based on the examination of 50 microscopic fields at 1000x magnification, i.e., 3,000 erythrocyte cells, and flow cytometry is programmed to analyze 50,000 cells. New microscopists were involved in this experiment to diversify the interpretation and human skills compared to previous experiments. In addition, we have conducted additional analyses to ascertain the sensitivity and specificity performance of the MALARIS system at threshold values of 4% and 10%, focusing on the context of imported malaria diagnosis. These analyses compare the MALARIS system’s outcomes against the Miller reticle technique and flow cytometry.

## Results

### Manual techniques as a source of variability for the parasitemia

**Fig 2:**
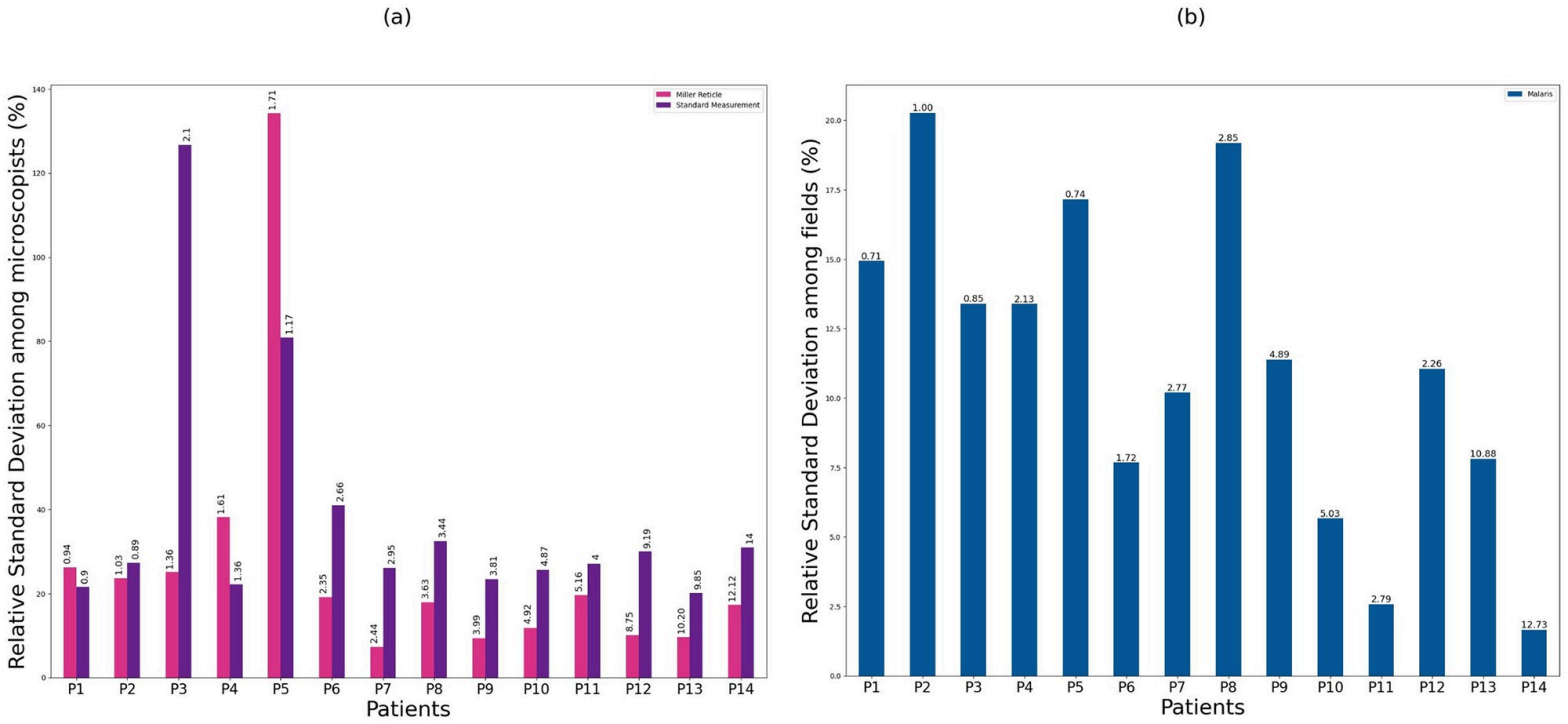
(a) Comparison of the relative standard deviation (RSD) among microscopists, ordered by increasing parasitemia, between two manual techniques, including 10 measurements randomly selected from 10 microscopists, resulted in a mean RSD of 26.40% for the Miller reticle method and 38.22% for the standard measurement method. (b) Distribution of RSD among fields, including the MALARIS estimation on 10 field images. Above each histogram, the measured parasitemia value for each patient, arranged in ascending order of parasitemia, is displayed.

Parasitemia estimation in 14 patients by the three different methods led to a comparative analysis between manual techniques and our MALARIS system. In Fig 2a, wecan observe more significant interhuman variability with the standard WHO procedure (mean RSD of 38.22% and median of 27.22%) based on 10 randomly selected measurements from the 10 microscopists, as opposed to the Miller reticle (mean RSD of 26.40% and median of 18.54%). In Fig 2b, the MALARIS system demonstrates a lower RSD (mean of 11.21% and median of 10.98%). Significant variation is observed in the standard measurement, with an RSD exceeding 100% for patient P3, similarly for patient P5 with the Miller reticle. Note that this indirect comparison makes it possible to analyze the three methods on the same data, but the relative standard deviations are calculated differently.

The confidence intervals displayed in S1 Fig provide estimates for each patient individually, offering insights into the intra-patient variability for each technique. These intervals illustrate both the variations between different techniques and the variations observed among individual patients. The confidence intervals for the manual standard and Miller reticle-assisted techniques exhibit significant variability as parasitemia increases. On the other hand, it demonstrates close average estimates among all three techniques, except for one patient (P12) where the MALARIS system underestimates the parasitemia.

### Parasitemia repeatability using MALARIS: The impact of the number of fields

The repeatability of the parasitemia count by the MALARIS system on the 14 patients allowed us to observe the impact of the number of fields on the quantification of *Plasmodium falciparum*-infected RBCs for each patient. For each patient with 10 fields, this led to a total of 45 pairs for k = 1, 630 pairs for k = 2, 2,100 pairs for k = 3, 1,575 pairs for k = 4, and 126 pairs for k = 5. The pairs represent different combinations of k fields for which the parasitemia is estimated.

**Fig 3:**
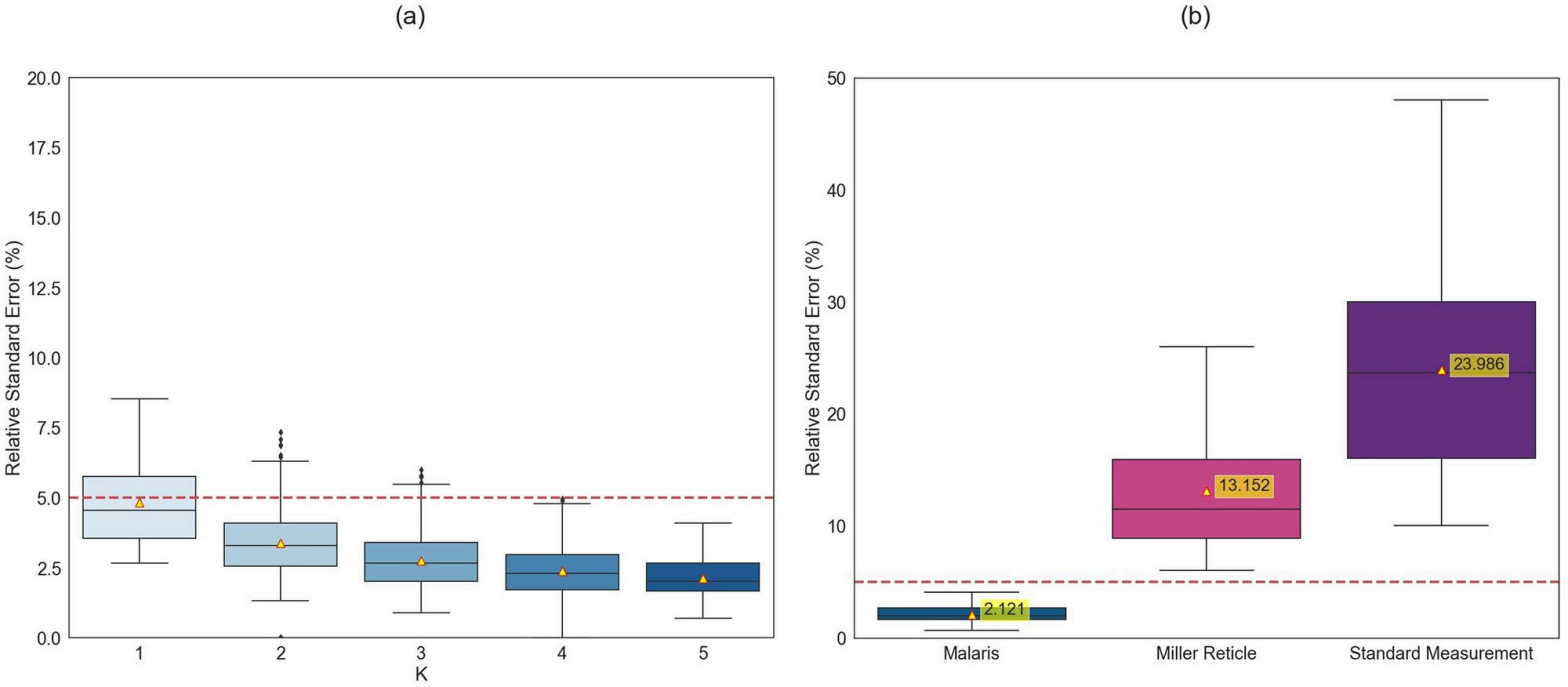
Repeatability of the parasitemia automatic count by the MALARIS system was assessed on 14 patients with estimated parasitemia from 0.7% to 12.72% and a median value of 2.01%. The relative standard error rate (RSE) (%) was represented to evaluate the repeatability of k combinations (1 to 5) using 10 thin blood smear images (a). Additionally, a comparison was made with the Miller reticle and Standard manual count (b). The yellow triangles represent the mean value of each pair of measurements.

For each combination of field images, the MALARIS system detected and classified an average of 1,000 blood cell images per field. The boxplots in Fig 3a show the distribution of the relative standard error (RSE) according to the average parasitemia calculated per combination of 1 to 5 field images. Notably, taking an image of a field at 500x, sending it to the system, and analyzing it takes a total of 80–90 seconds on average for more than 1,000 blood cell images.

In addition, Fig 3b reports the results obtained on 5 fields (blue boxplot) compared with the parasitemia calculated by the microscopists (per pair of two microscopists) including the Miller reticle (pink boxplot) and the standard measurement (purple boxplot). The results show a very low RSE for the MALARIS system compared with other methods (mean RSE of 2.12% with a range between 0.70% and 4.10%). The Miller reticle also shows a low RSE (mean RSE value of 13.15% with a range between 6.05% and 26.01%) but higher than that of our system. The standard measurement, which is the most variable of the methods, shows a high variation (mean RSE of 23.98% with a range between 10.04% and 48.06%).

### MALARIS vs. manual and semiautomatic methods, including Miller reticle and flow cytometry

Firstly, we evaluated whether 5,000 RBC images were sufficient for our system, as demonstrated previously. We confirmed these results by studying the variability, but this time on a larger sample size of 102 patients. For this purpose, we investigated the variability of the 5-field combinations for these 102 patients by calculating the relative standard error (RSE) for the 126 possible pairs of k = 5 microscopic fields (S3 Fig). The results revealed an average RSE of 4.94% and a median of 3.89%. The minimum RSE observed was 0.4%, while the maximum reached 22.86%. These findings support the results obtained from the analysis of the 14 patients, where the mean RSE was below 10%, specifically at 2.12%. Our findings demonstrate that regardless of the combination of 5 images, the measurements consistently fall within a limited range.

**Fig 4:**
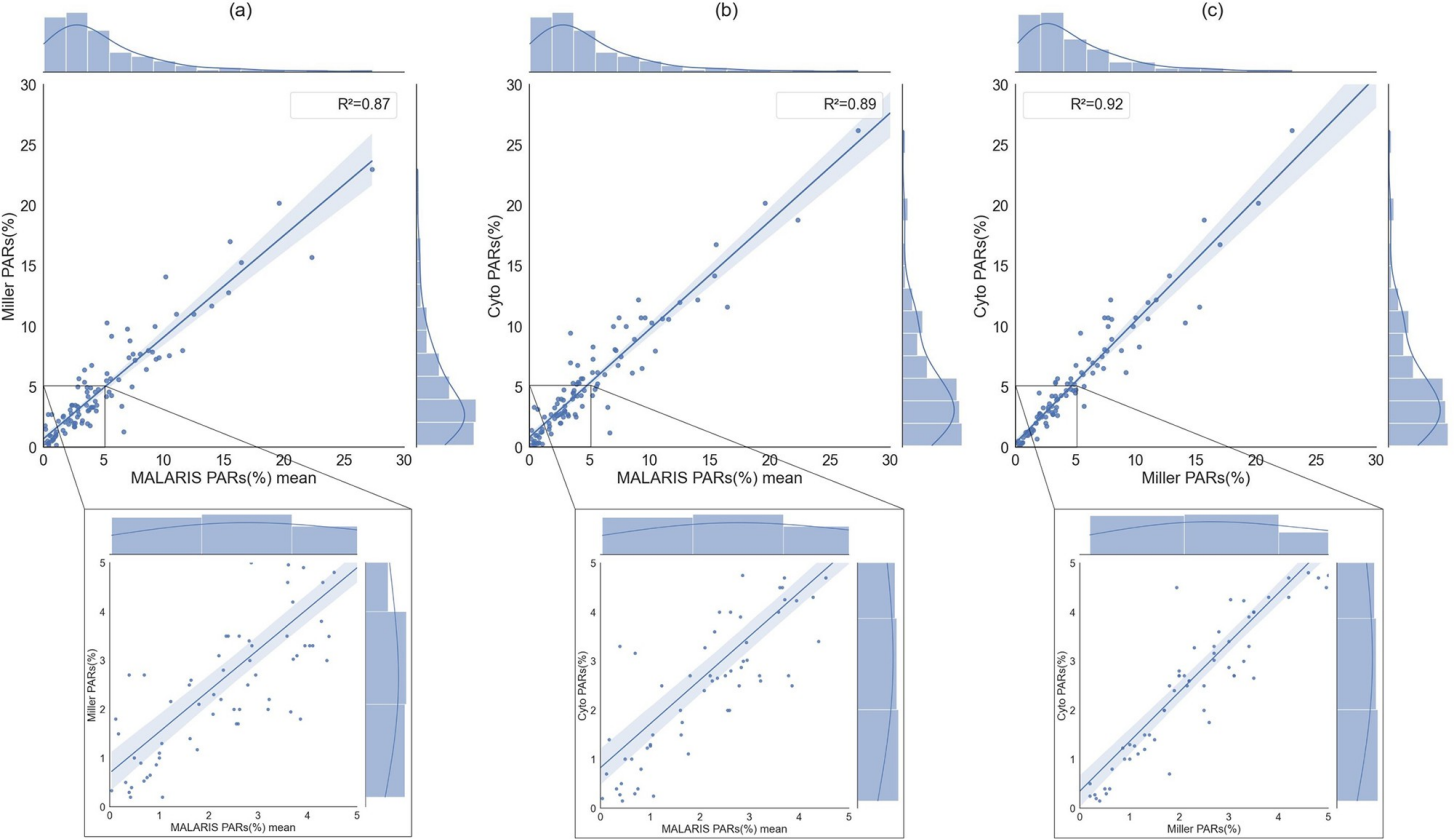
Comparison of regression plots for parasitemia estimation. MALARIS (x-axis) vs. Miller reticle (y-axis) (a), MALARIS (x-axis) vs. flow cytometry (y-axis) (b), and Miller reticle (x-axis) vs. flow cytometry (y-axis) (c). Below the plots, there is a specific focus on the values of parasitemia between 0 and 5%.

To further evaluate the methods for parasitemia estimation, we compared the results obtained for each technique on 102 new patients and calculated their R^2^ values to estimate the concordance between the results obtained from each method. Our MALARIS system showed a strong correlation coefficient (R^2^ = 0.87) with the parasitemia obtained using the Miller reticle (Fig 4a). Similarly, we also observed a strong correlation with flow cytometry (R^2^ = 0.89) as shown Fig 4b. Additionally, we noted a strong correlation (R^2^ = 0.92) between the Miller reticle method and flow cytometry measurements (Fig 4c). At threshold of 4%, both MALARIS with Miller and MALARIS with flow cytometry demonstrate high sensitivity. Specifically, MALARIS with Miller exhibits a sensitivity of 84.1%, while MALARIS with flow cytometry surpasses it with a sensitivity of 93.2%. When the threshold is increased to 10%, the sensitivity values for both methods remain relatively high. MALARIS with Miller achieves a sensitivity of 83.3%, and MALARIS with flow cytometry maintains a sensitivity of 91.7%. At threshold of 4%, MALARIS with Miller displayed slightly higher specificity (86.2%) compared to MALARIS with flow cytometry (77.6%). For a threshold of 10%, MALARIS with Miller exhibited an exceptionally high specificity of 97.8%. MALARIS with flow cytometry, at this threshold, maintained a commendable specificity of 93.3% (S2 Table). Additionally, our evaluation delved into the performance of the reference methods themselves. When we compared Miller reticle with flow cytometry, some interesting trends emerged. At threshold of 4%, Miller reticle paired with flow cytometry showed excellent sensitivity at 97.8%, with specificity at 82.5%. This highlights the robustness of this combined approach in correctly identifying patients with parasitemia levels above 4% while keeping the rate of false positives relatively low. For a threshold of 10%, the Miller reticle and flow cytometry combination maintained high sensitivity at 91.7% and high specificity at 93.3%.

In addition, the Bland-Altman plots in S2 Fig provide a visual representation of the agreement between the three techniques for parasitemia measurement. In each plot, the red line, representing the mean difference, is observed to be centered around 0. This indicates a good level of agreement between the two techniques being compared. The consistent alignment of the red lines around 0 across all three plots demonstrates the consistency and agreement in parasitemia estimations obtained using different techniques. These findings reaffirm the reliability and accuracy of the measurements.

## Discussion and conclusions

The main contributions of this study are (1) the repeatability of our MALARIS system for the determination of the optimal number of fields required at 500x magnification for the parasitemia estimation; (2) highlighting the limitations of current manual techniques; and (3) the evaluation and comparison of three techniques including MALARIS, semiautomatic flow cytometry and Miller reticle.

This study is the first to evaluate and compare the performance of an AI-based system in measuring parasitemia from thin blood smears for routine *P*. *falciparum* infections using both manual and semiautomated methods (S1 Table). To accomplish this, we conducted a retrospective analysis of imported malaria in a clinical setting. Despite the critical role of parasitemia in assessing disease severity and mortality risk, few studies have assessed the accuracy of calculation systems for this measure on a large dataset of patient data. Our system provides a quick and easy estimation of parasitemia within 10 minutes (using 10 field images at 500x magnification), enabling physicians to promptly determine the infection’s severity and adjust management strategies accordingly.

We conducted experiments using three different techniques, ranging from the most cost-effective approach of using a smartphone camera combined with our proposed AI-based system, to microscopy assisted by the Miller reticle, and extending to more advanced techniques like flow cytometry. Our proposed MALARIS can analyze images of thin blood smears at 500x magnification. The second technique employed the Miller reticle, which was validated internally in our laboratory, and a third technique based on DNA markers, utilizing commercial flow cytometry. In our experiments, we investigated the impact of the number of fields on the variation in parasitemia calculation by capturing images from 10 fields of 14 patients. Our findings demonstrated that a small variation in the average relative standard error (RSE) (<10%) for 5 field images allows for an estimation of parasitemia (Fig 3), which is equivalent to more than 5,000 images of red blood cells analyzed by our system. This is in line with WHO recommendations.

In addition, we demonstrated that interhuman variability in manual microscopy using the gold standard measurement was significant (with an average relative standard deviation of 38.22% between microscopists).

To demonstrate the effectiveness of our model, we compared it with another semiautomated method using flow cytometry (Fig 4). Our MALARIS system demonstrated a stronger correlation (R^2^ = 0.89) with the results obtained using flow cytometry compared to the Miller reticle method (R^2^ = 0.87), although both correlations remain relatively close. This indicates that the method comparisons remain favorable, whether based on microscopy or flow cytometry. These findings highlight the promising results of a cost-effective system such as MALARIS in estimating parasitemia and its compatibility with different measurement techniques.

We designed a promising MALARIS pipeline using 500x magnification images of thin blood smears. It enables accurate estimation of parasitemia within a time frame compatible with clinical management of the patient. The slides used in this study were not selected on the basis of their quality, and although we have reinforced model training with uninfected images of RBCs containing staining precipitations or artifacts, additional staining problems or artifacts may occur, especially on thin smears produced in endemic regions [14]. Slide staining conditions may be of poorer quality than in the context of imported malaria such as ours. Even if MALARIS presents a higher level of suitability in diagnosing imported cases of malaria and measuring parasitemia, there will be a need for complementary studies in endemic countries. Our single-center study limits the scope of the results obtained, in particular the use of different reagents and staining intensity, have not been tested on an operational multicenter scale. The impact of color intensity could be controlled by performing manual staining and using a great diversity of slides in our study, but it is likely that a color normalization technique to standardize colors and textures would be needed on a larger scale [25]. The system may have other limitations for low parasitemia levels (<1%) as 5,000 images may not be sufficient. To overcome this limitation, flow cytometry, which is more efficient and sensitive for low parasitemia counting between 0.1% and 1% [26] could be used. However, the high cost, the need to have providers trained in its use and the low availability of flow cytometry, particularly in areas with scarce resources, limit its use in this indication. Frugal AI-based systems offer significant potential to improve the overall performance of this diagnostic. Additionally, capturing images of microscopic fields at 500x magnification, which is simple and fast, allows for remote experts to confirm diagnoses. AI clearly has the potential to dramatically improve the diagnosis and quality of patient care in this important global health challenge.

Moving forward, our future endeavors with the MALARIS framework encompass the integration of species identification capabilities. This will undoubtedly contribute to refining diagnostic precision. Furthermore, we intend to embark on a comprehensive multicentric evaluation, spanning regions of varying endemicities and encompassing higher parasitemia levels and diverse conditions. By subjecting the system to such rigorous assessments, we aim to establish its robustness and reliability across different laboratory settings. Ultimately, our vision involves transforming MALARIS into a versatile computer-assisted tool, capable of remotely initiating preliminary analyses on microscopy specimens, including *P*. *falciparum*-infected RBCs and parasitemia measurements, thereby offering invaluable support in guiding patient care decisions.

## Supporting information

S1 Text Estimation of malaria parasitemia. (DOCX)

S2 Text Relative Standard Deviation (RSD). (DOCX)

S3 Text Relative Standard Error (RSE). (DOCX)

S1 Table Comparison of the techniques used for the parasitemia estimation. (DOCX)

S2 Table Evaluation of sensitivity and specificity at threshold of 4% and 10%. (DOCX)

S1 Fig Comparison of parasitemia estimates and confidence intervals in 14 patients. Malaris vs. Miller Reticle vs. Standard Measurement. Each circle is centered at the mean value of the corresponding sample.
(TIF)

S2 Fig Bland-Altman plots of the techniques used for the parasitemia estimation. (a) Comparison between MALARIS and Miller reticle. (b) Comparison between MALARIS and flow cytometry. (c) Comparison between the Miller reticle and flow cytometry. The plots display the difference between the measurements against the average of the two measurements, with the red line representing the mean difference.
(TIF)

S3 Fig Repeatability of the parasitemia automatic count by the MALARIS system was evaluated on 102 patients with ordered parasitemia from 0.03% to 27.32%, and a median value of 3.63%. The repeatability was assessed using the relative standard error rate (RSE) (%) by analyzing multiple combinations of 5 thin blood smear images, resulting in a total of 126 pairs. The RSE (%) was employed as a measure to quantify the variability and assess the repeatability of the measurements across different field combinations. The average RSE is 4.94%, and the median is 3.89%. The red dashed line represents the 10% RSE threshold.
(TIF)
