# Supplementary material for: Evaluating Plasmodium falciparum automatic detection and parasitemia estimation: A comparative study on thin blood smear images
Source: PLoS One. 2024 Jun 3;19(6):e0304789. doi: 10.1371/journal.pone.0304789 (PMC11146722; doi:10.1371/journal.pone.0304789)
Supplement: S1 Text — (DOCX) [file pone.0304789.s001.docx]

##### **S1 Text. Estimation of malaria parasitemia.**

Parasitemia can be assessed by several methods, including Miller cell, standard measurement and flow cytometry. For this task the formula is as follows:

$$Parasitemia \left( \% \right)=\frac{Number of infected RBCs}{Total number of RBCs}\times100$$

(1)

If a red blood cell has several parasites, it is counted as one infected RBC.
