# Supplementary material for: Evaluating Plasmodium falciparum automatic detection and parasitemia estimation: A comparative study on thin blood smear images
Source: PLoS One. 2024 Jun 3;19(6):e0304789. doi: 10.1371/journal.pone.0304789 (PMC11146722; doi:10.1371/journal.pone.0304789)
Supplement: S2 Text — (DOCX) [file pone.0304789.s002.docx]

##### **S2 Text. Relative standard deviation (RSD).**

$$RSD\left( \% \right)=\frac{\mathrm{SD}}{X̄}\times100$$

(2)

##### RSD (Relative Standard Deviation) is expressed as a percentage and defined as the standard deviation of measurements obtained by different pairs of fields or microscopists (SD), divided by the average of the parasitemia measurements (X̄). The relative standard deviation serves as a measure of the degree of variability between different parasitemia measurements. A smaller RSD indicates higher agreement and consistency between measurements of parasitemia, while a larger RSD suggests greater variability and inconsistency.
