# Supplementary material for: Evaluating Plasmodium falciparum automatic detection and parasitemia estimation: A comparative study on thin blood smear images
Source: PLoS One. 2024 Jun 3;19(6):e0304789. doi: 10.1371/journal.pone.0304789 (PMC11146722; doi:10.1371/journal.pone.0304789)
Supplement: S3 Text — (DOCX) [file pone.0304789.s003.docx]

##### **S3 Text. Relative standard error (RSE).**

$$RSE\left( \% \right)=\frac{\mathrm{SE}}{X}\times100$$

(3)

RSE (Relative Standard Error) is the relative standard error expressed as a percentage, SE is the standard error of the differences between the two combinations of measurements, and X is the mean of the two combinations of measurements. The relative standard error measures the precision of the difference between two combinations of fields. A smaller RSE indicates greater precision and less variability in the difference between the two combinations of measurements. A larger RSE indicates less precision and more variability in the difference.
