# Supplementary material for: Evaluating Plasmodium falciparum automatic detection and parasitemia estimation: A comparative study on thin blood smear images
Source: PLoS One. 2024 Jun 3;19(6):e0304789. doi: 10.1371/journal.pone.0304789 (PMC11146722; doi:10.1371/journal.pone.0304789)
Supplement: S1 Table — (DOCX) [file pone.0304789.s004.docx]

**S1 Table. Comparison of the techniques used for the parasitemia estimation.**

| **Technique** | **Type** | **Technique** | **Magnification​** | **Expertise** | **Number of cells** | **Cost** | **Time consumption** |
| --- | --- | --- | --- | --- | --- | --- | --- |
| **MALARIS** | Semiautomatic | Microscopy | 500x | No | 5,000 to 10,000 | Low | Low |
| **Standard measurement** | Manual | Microscopy | 1000x | Yes | 20,000 | Low | High |
| **Miller cell** | Manual | Microscopy | 1000x | Yes | 3,000 | Low | High |
| **Flow cytometry** | Semiautomatic | DNA marking | - | Yes | 50,000 | High | Low |
