# Supplementary material for: Evaluating Plasmodium falciparum automatic detection and parasitemia estimation: A comparative study on thin blood smear images
Source: PLoS One. 2024 Jun 3;19(6):e0304789. doi: 10.1371/journal.pone.0304789 (PMC11146722; doi:10.1371/journal.pone.0304789)
Supplement: S2 Table — (DOCX) [file pone.0304789.s005.docx]

**S2 Table.** **Evaluation of sensitivity and specificity at Limit of Detection (LOD) values of 4% and 10%.**

| **Techniques** | **Sensitivity (%)** | | **Specificity (%)** | | |
| --- | --- | --- | --- | --- | --- |
| **LOD** | 4% | 10% | | 4% | 10% |
| **MALARIS with Miller reticle** | 84.1 | 83.3 | | 86.2 | 97.8 |
| **MALARIS with flow cytometry** | 93.2 | 91.7 | | 77.6 | 93.3 |
| **Miller reticle with flow cytometry** | 97.8 | 91.7 | | 82.5 | 93.3 |

$Sensitivity=\frac{\mathrm{TP}}{TP+FN}$ $Specificity=\frac{\mathrm{TN}}{TN+FP}$

These results represent the sensitivity and specificity performance of the MALARIS system using the Miller reticle and flow cytometry as the reference methods. Sensitivity measures the system's ability to correctly identify patients with malaria who have parasitemia levels above the specified LOD threshold, while specificity measures the system's ability to correctly identify patients with parasitemia levels below the LOD threshold. Additionally, we evaluated the performance of the reference methods themselves.

- True Positive (TP):
  - TP represents the number of cases where both the MALARIS system and the Miller technique/flow cytometry correctly identified the patient as having parasitemia levels above the specified LOD threshold.
- False Negative (FN):
  - FN represents the number of cases where the MALARIS system had a positive result (above LOD), but the Miller technique/flow cytometry had a negative result (below LOD).
- True Negative (TN):
  - TN represents the number of cases where both the MALARIS system and the Miller technique/flow cytometry correctly identified the patient as not having parasitemia levels above the specified LOD threshold.
- False Positive (FP):
  - FP represents the number of cases where the MALARIS system had a positive result (above LOD), but the Miller technique/ flow cytometry had a negative result (below LOD).
