# Supplementary figures and images for: Evaluating Plasmodium falciparum automatic detection and parasitemia estimation: A comparative study on thin blood smear images
Source: PLoS One. 2024 Jun 3;19(6):e0304789. doi: 10.1371/journal.pone.0304789 (PMC11146722; doi:10.1371/journal.pone.0304789)

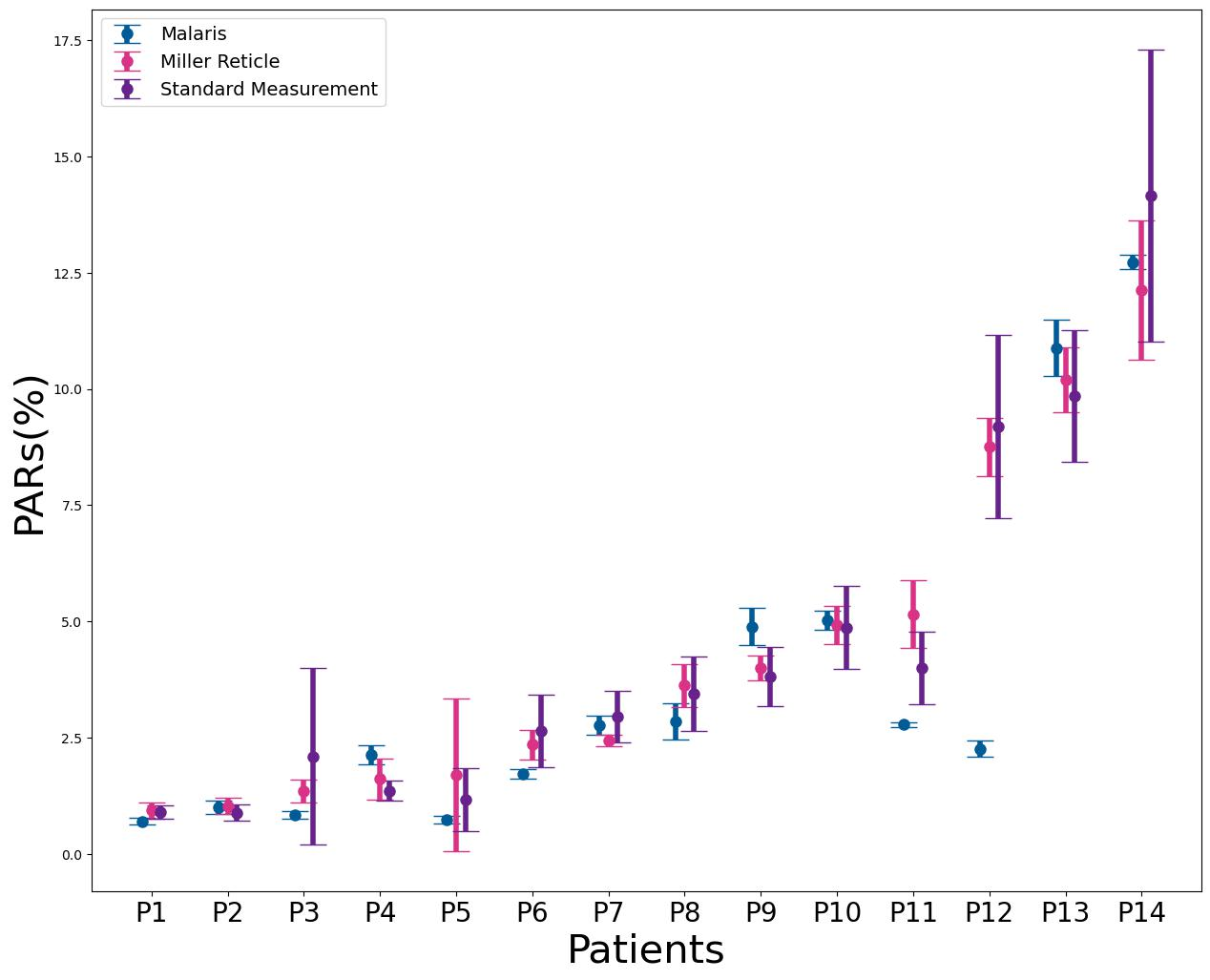

Supplement: S1 Fig — Malaris vs. Miller Reticle vs. Standard Measurement. Each circle is centered at the mean value of the corresponding sample. (TIF) [file pone.0304789.s006.tif]

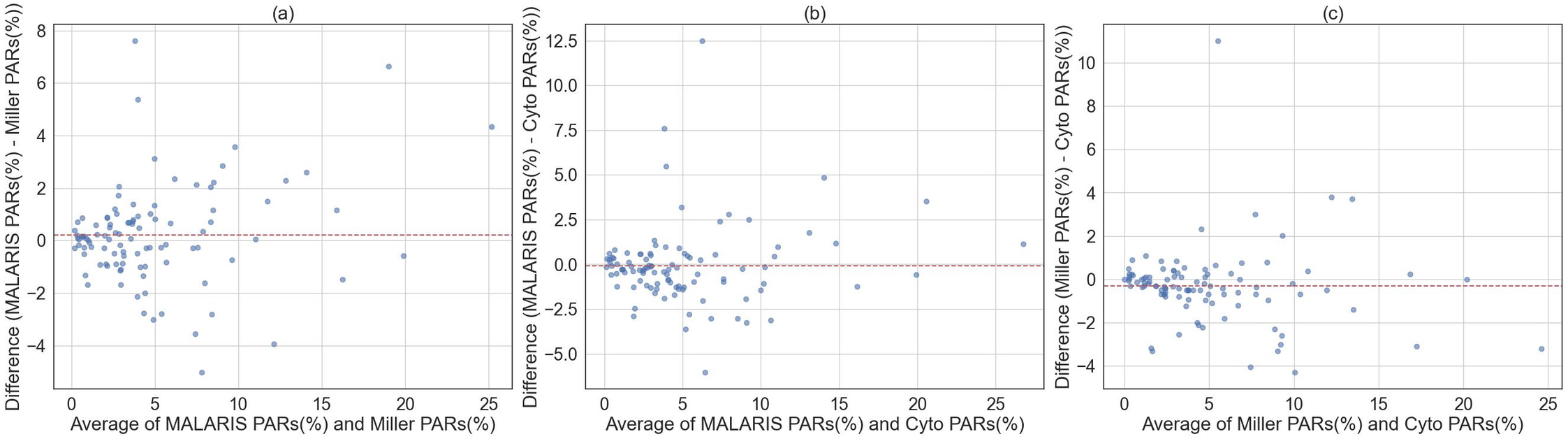

Supplement: S2 Fig — (a) Comparison between MALARIS and Miller reticle. (b) Comparison between MALARIS and flow cytometry. (c) Comparison between the Miller reticle and flow cytometry. The plots display the difference between the measurements against the average of the two measurements, with the red line representing the mean difference. (TIF) [file pone.0304789.s007.tif]

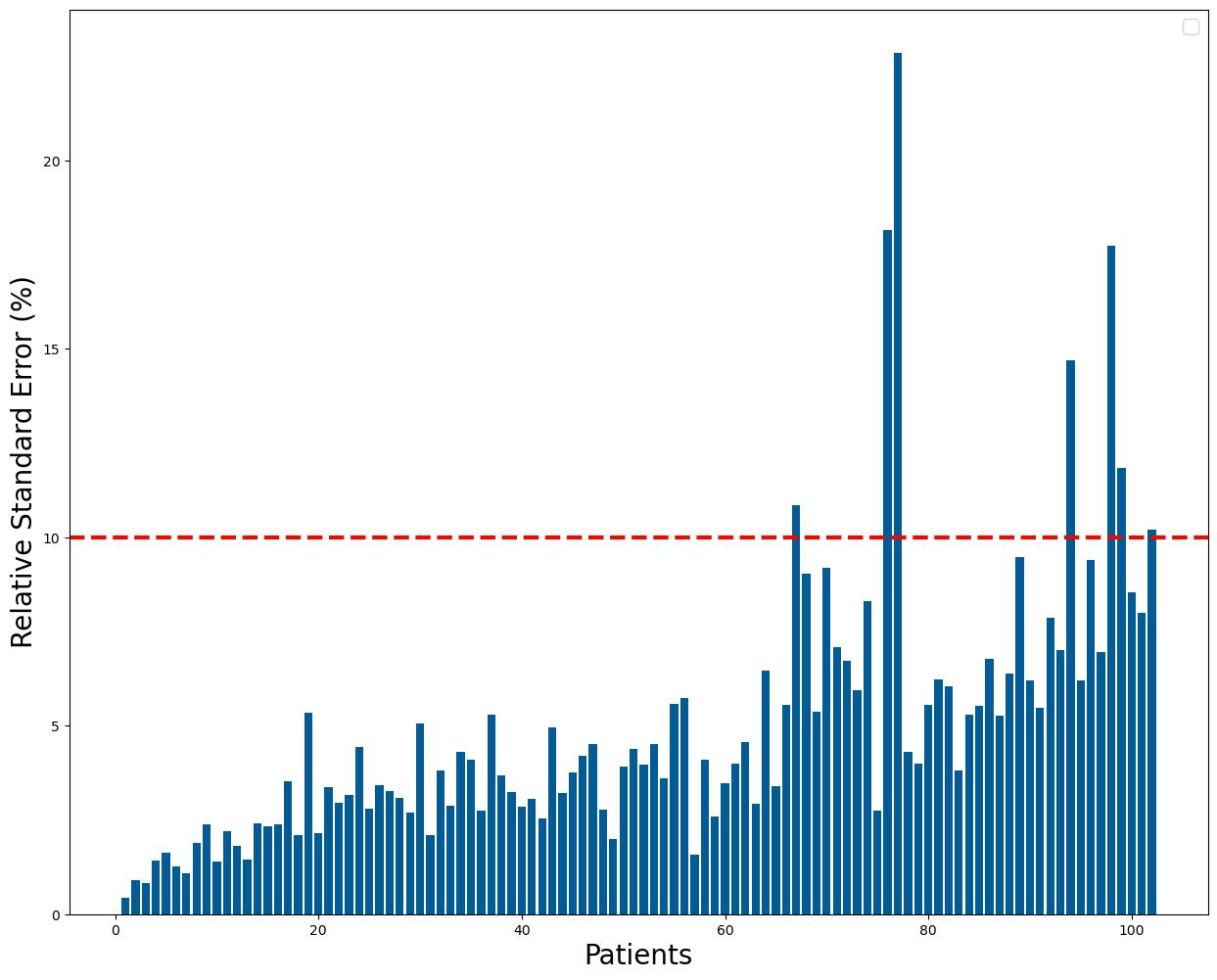

Supplement: S3 Fig — The repeatability was assessed using the relative standard error rate (RSE) (%) by analyzing multiple combinations of 5 thin blood smear images, resulting in a total of 126 pairs. The RSE (%) was employed as a measure to quantify the variability and assess the repeatability of the measurements across different field combinations. The average RSE is 4.94%, and the median is 3.89%. The red dashed line represents the 10% RSE threshold. (TIF) [file pone.0304789.s008.tif]
